# Proteomic identification of heat shock protein 90 as a candidate target for p53 mutation reactivation by PRIMA-1 in breast cancer cells

**DOI:** 10.1186/bcr1290

**Published:** 2005-07-27

**Authors:** Abdur Rehman, Manpreet S Chahal, Xiaoting Tang, James E Bruce, Yves Pommier, Sayed S Daoud

**Affiliations:** 1 Department of Pharmaceutical Sciences, Washington State University, Pullman, WA, USA; 2 Pharmacology and Toxicology Graduate Program, Washington State University, Pullman, WA, USA; 3 Department of Chemistry, Washington State University, Pullman, WA, USA; 4 Laboratory of Molecular Pharmacology, Center for Cancer Research, National Cancer Institute, National Institutes of Health, Bethesda, MD, USA

## Abstract

**Introduction:**

A loss of p53 function resulting from mutation is prevalent in human cancers. Thus, restoration of p53 function to mutant p53 using small compounds has been extensively studied for cancer therapy. We previously reported that PRIMA-1 (for 'p53 reactivation and induction of massive apoptosis') restored the transcriptional activity of p53 target genes in breast cancer cells with a p53 mutation. By using functional proteomics approach, we sought to identify molecular targets that are involved in the restoration of normal function to mutant p53.

**Methods:**

PRIMA-1 treated cell lysates were subjected to immunoprecipitation with DO-1 primary antibody against p53 protein, and proteins bound to p53 were separated on a denaturing gel. Bands expressed differentially between control and PRIMA-1-treated cells were then identified by matrix-assisted laser desorption ionization-time-of-flight spectrometry. Protein expression in whole cell lysates and nuclear extracts were confirmed by Western blotting. The effect of combined treatment of PRIMA-1 and adriamycin in breast cancer cells was determined with a cytotoxicity assay *in vitro*.

**Results:**

PRIMA-1 treated cells distinctly expressed a protein band of 90 kDa that was identified as heat shock protein 90 (Hsp90) by the analysis of the 90 kDa band tryptic digest. Immunoblotting with isoform-specific antibodies against Hsp90 identified this band as the α isoform of Hsp90 (Hsp90α). Co-immunoprecipitation with anti-Hsp90α antibody followed by immunoblotting with DO-1 confirmed that p53 and Hsp90α were interacting proteins. PRIMA-1 treatment also resulted in the translocation of Hsp90α to the nucleus by 8 hours. Treatment of cells with PRIMA-1 alone or in combination with adriamycin, a DNA-targeted agent, resulted in increased sensitivity of tumor cells.

**Conclusion:**

The studies demonstrate that PRIMA-1 restores the p53-Hsp90α interaction, enhances the translocation of the p53-Hsp90α complex and reactivates p53 transcriptional activity. Our preliminary evidence also suggests that PRIMA-1 could be considered in combination therapy with DNA-targeted agents for the treatment of breast cancer, especially for tumors with aberrant p53 function.

## Introduction

Many clinical studies have shown that mutations in *p53 *are a strong predictor of relapse and are associated with resistance to several therapeutic regimens [1,2]. Studies in our laboratories and others, for example, showed that mutations in *p53 *in human tumor cells were correlated with decreased sensitivity to DNA-damaging agents [3-6]. An improved understanding of the relationship between *p53 *and chemosensitivity might therefore lay the groundwork for new cancer therapies. To understand this relationship better, we recently used a pharmacogenomic approach with complementary DNA microarrays to characterize gene expression profiles of cells containing wild-type p53 (*p53*^+/+^) and those containing an isogenic p53 knockout counterpart (*p53*^-/-^) after treatment with topotecan, a specific topoisomerase I inhibitor and a DNA-targeted agent [7]. About 10% of the transcripts detected were differentially expressed in the *p53*^+/+ ^cells in response to topotecan, whereas only 1% of the transcripts changed in the *p53*^-/- ^cells [7]. These data clearly showed the broad effect of p53 on the transcriptional response to DNA damage, which can lead to growth arrest or apoptosis.

Given that *p53 *is the most commonly mutated gene in human cancers and that more than 50% of breast tumors are defective in *p53 *[8-10], extensive research efforts are centered on restoring normal function to mutant p53 to promote tumor suppression. This effort includes the use of modifying peptides [11,12], antisense oligonucleotides [13] and small molecules [14,15]. Unfortunately, the problem of *in vivo *delivery and lack of selectivity to tumor cells has limited the practical application of most of these efforts. Recently, PRIMA-1 has emerged from an *in vitro *screen of small molecules that reactivate the transcriptional activity of mutant p53 [16]. PRIMA-1 has the capability of restoring the transcriptional transactivation function to mutant p53 *in vitro *and *in vivo *with subsequent tumor regression. PRIMA-1 also has the ability to trigger apoptosis in tumor cells as a function of its mutant p53 reactivation response [17]. We reported recently [18] that PRIMA-1 (in a effect dependent on both dose and time) restored the transcriptional activity of *p53 *target genes such as p21^*Waf1*/*cip1 *^in breast cancer cells possessing a *p53 *mutation. However, the exact molecular mechanisms for mutant p53 reactivation by PRIMA-1 are not yet determined. A direct interaction between PRIMA-1 and p53 has not yet been demonstrated. It is possible that PRIMA-1 affects cellular chaperones, resulting in the refolding of mutant p53. Alternatively, PRIMA-1 may block complex formation between mutant p53 and p73, leading to the release of active p73, which triggers proapoptotic target genes [15]. To identify the possible molecular candidates of mutant p53 reactivation by PRIMA-1 in breast tumor cells, in this study we used tools available for a functional proteomics approach.

Our study indicates that the restoration of the transcriptional transactivation of *p53 *target genes such as p21^*Waf1*/*cip1 *^is dependent on p53 and that the α isoform of heat shock protein 90 (Hsp90α) is associated with mutant p53 reactivation by PRIMA-1. As a result of refolding of mutant p53 by Hsp90, we show that both p53 and Hsp90α are translocated to the nucleus of the tumor cells for the activation of p53 target genes. The use of PRIMA-1 to reactivate mutant p53 may therefore be considered further as an approach for adjuvant chemotherapy in the treatment of breast tumors, especially in cancers with aberrant p53 function.

## Materials and methods

### Drugs and materials

PRIMA-1 (NSC-281668) was obtained from the Drug Synthesis and Chemistry Branch, National Cancer Institute (Bethesda, MD); pifithrin-α (PFTα) and doxorubicin (adriamycin hydrochloride) were purchased from Biomol Research Inc. (Plymouth Meeting, PA). Primary antibodies against p53 and p21 were purchased from Santa Cruz Biotechnology (Santa Cruz, CA); Hsp 90 primary antibodies were from Stressgen (Victoria, BC, Canada). The goat anti-mouse, anti-rat and anti-rabbit secondary antibodies labeled with IRDye™ 38 were purchased from LI-COR, Inc. Biosciences (Lincoln, NE) or with Alex680 from Molecular Probes, Inc. (Eugene, OR). All other chemicals were of reagent grade.

### Cell and culture conditions

The human MCF-7 breast carcinoma cells (*p53*^+/+^), MDA-MB-231 and GI-101A (*p53 *mutant) were routinely maintained in monolayer cultures in RPMI-1640 medium (Invitrogen, Inc., Carlsbad, CA) supplemented with 10% fetal bovine serum (Hyclone, Logan, UT) as reported previously [3]. As we reported previously [18], p53 protein in GI-101A cells contains mutations at Y236C, A278P and R72P, whereas p53 protein in MDA-231 cells contains mutations at A278P, R280K and M385T. Exponentially growing cultures at 80% confluence were used in all experiments. For cytotoxicity assays, cells from exponentially growing cultures were plated in 24-well tissue culture plates in RPMI-1640 medium plus 10% fetal calf serum at about 10^4 ^cells per well, and the IC_50 _doses of PRIMA-1 in MDA-231 and GI-101A cells were determined as reported previously [18]. For drug combination studies, we used 100 μM PRIMA-1 and 0.2 μM adriamycin. Both cell lines were treated with the following drug sequence: A3, cells were treated with adriamycin for 3 hours; A24, cells were treated with adriamycin for 24 hours; P24, cells were treated with PRIMA-1 for 24 hours; AP24, cells were treated with both adriamycin and PRIMA-1; and A3P24, cells were treated with adriamycin for 3 hours followed by the removal of adriamycin-containing medium, washing with fresh medium and then incubation with PRIMA-1 for 24 hours. Each treatment was performed in quadruplicate wells in two independent experiments. The wells were washed twice with prewarmed medium, followed by the addition of 2 ml of drug-free medium. The plates were then incubated for a further 3 days. The surviving fraction of cells was determined with the crystal violet assay, as reported previously [19]. The precision of this method with quadruplicate determinations is 10% (SD). The data were analyzed with Student's two-tailed *t*-test; *P *< 0.01 was considered statistically significant.

### Immunoblotting and immunoprecipitation

Cells were incubated with 0 or 100 μM PRIMA-1 for 0, 2, 4 or 8 hours, then washed twice with PBS (pH 7.4) and harvested. Harvested cells were either used to prepare whole cell lysates or for subcellular fractionation studies. Cell lysates were prepared as described previously [7]. For immunoblotting, 20 μg protein samples were separated by SDS-PAGE (4 to 20% polyacrylamide gradient gel) and transferred on nitrocellulose (Millipore, Bedford, MA). The membrane was developed in accordance with a protocol provided by LI-COR, Inc. Biosciences using anti-β-actin, anti-p53 and anti-p21 primary antibodies (Santa Cruz Biotechnology) or anti-Hsp90α and anti-Hsp90β primary antibodies (Stressgen). The goat anti-mouse, anti-rat and anti-rabbit secondary antibodies labeled with IRDye™ 38 (ex/em: 774/800 nm; LI-COR) or with Alexafluor^® ^680 (ex/em: 680/707 nm; Molecular Probes) were used. The reactive bands were revealed and detected with the Odyssey™ Infrared Imaging System (LI-COR, Inc.).

For immunoprecipitation studies, 10^7 ^MDA-MB-231 or GI-101A cell cultures were grown in T-150 tissue culture flasks. Cultures were treated with 100 μM PRIMA-1 for 4 hours. Cleared cell lysates (total 1 mg of protein) were immunoprecipitated either with 5 μg of DO-1 anti-p53 monoclonal antibody or 5 μg of anti-Hsp90α monoclonal antibody in a co-immunoprecipitation assay in accordance with the protocol provided by eBiosciences, Inc. (San Diego, CA) using Protein A or G-plus agarose beads (Santa Cruz Biotechnology). After SDS-PAGE (4 to 20% polyacrylamide), gels were either stained with Coomassie blue (Bio-Rad, Inc., Hercules, CA) or subjected to immunoblotting.

### In-gel enzymatic digestion and mass spectrometry

**Figure 1:**
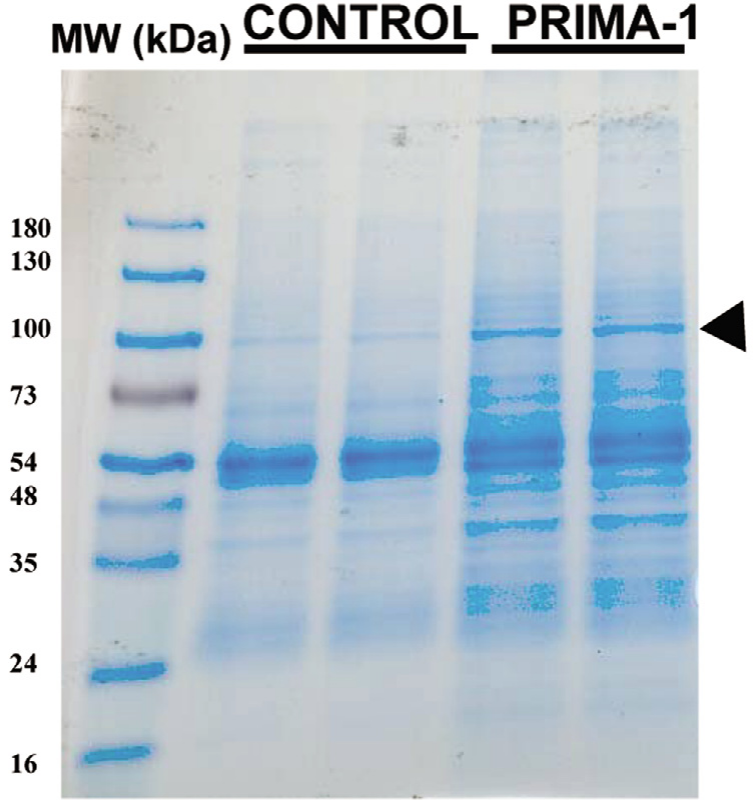
Coomassie blue-stained gel of proteins co-immunoprecipitated with DO-1 primary antibody from MDA-MB-231 cells. Cleared cell lysates were immunoprecipitated with DO-1 primary antibody directed against p53, washed, and resolved by SDS-PAGE (4 to 20% polyacrylamide). Two independent co-immunoprecipitated samples from untreated control (-) and cells treated for 4 hours with 100 μM PRIMA-1 (+) were loaded. The gels were stained with Coomassie blue. Molecular masses of protein size markers are indicated (MW). The arrowhead indicates the band of stained proteins excised for enzymatic digestion by trypsin and subsequent mass fingerprinting with matrix-assisted laser desorption ionization-time-of-flight mass spectrometry.

The protein bands were excised from the one-dimensional Coomassie blue-stained polyacrylamide gel shown in Fig. 1. After reduction and alkylation the protein bands were dehydrated in acetonitrile and dried. The bands were digested in the gel with an excess of sequencing-grade trypsin (Promega, Madison, WI). The digestion was performed overnight at 37°C. The resulting tryptic peptides were extracted from the gels, then desalted and concentrated with C_18 _ziptip (Millipore) before spotting for matrix-assisted laser desorption ionization-time-of-flight (MALDI-TOF) analysis.

An OmniFlex MALDI-TOF mass spectrometer (Bruker Daltonics, Billerica, MA) was used for peptide mass fingerprinting. Desalted peptide solution (1 μl) was mixed with 1 μl of matrix solution (α-cyano-4-hydroxycinnamic acid in 0.1% trifluoroacetic acid and 50% acetonitrile) and was then spotted directly on the MALDI target. All data used for protein identification and calibration were acquired in reflectron mode and averaged for 100 shots. These studies employed external mass calibration and used angiotensin I, corticotropin (ACTH) clip (1 to 17) and ACTH clip (18 to 39) peptides as calibrants. After spectral acquisition, the peak *m*/*z *values were extracted and used to search the Swiss Protein Database with the ProFound program [20] search engine. The database search was performed with an implementation of the following search parameters: taxonomy (*Homo sapiens*), enzyme (trypsin), missing cleavage, mass tolerance (0.24 Da), modification (carbamidomethyl for cysteine and oxidation for methionine) and database (NCBRnr).

### Subcellular fractionation and immunocytochemistry

To determine the nuclear translocation of p53 and Hsp90α, cells treated with PRIMA-1 were subjected to nuclear isolation with the FOCUS cytoplasmic and nuclear protein extraction kit (Geno Technology, Inc., St. Louis, MO) in accordance with the manufacturer's instructions. A fraction of the isolated nuclear pellet was fixed in 4% paraformaldehyde in 1 × PBS to determine the intactness of nuclei by staining with 4',6-diamidino-2-phenylindole (DAPI). Fixed nuclei were mounted on SuperFrost-plus slides (Fisher Scientific, Pittsburgh, PA) with Prolong Gold antifade reagent with DAPI (Molecular Probes, Invitrogen Detection Technologies, Carlsbad, CA). Our nuclear preparation contained 99% intact nuclei. To determine the expression of p53, and Hsp90s in intact nuclear fractions, the nuclear pellet was lysed into 1 × SDS sample buffer and subjected to denaturing gel electrophoresis followed by immunoblotting as described above.

To confirm translocation of proteins into the nucleus, nuclear fractions were subjected to immunostaining for p53 and Hsp90α. Immunocytochemistry was performed in accordance with a previously described protocol, with modifications [21]. In brief, fixed nuclear fractions were suspended in antibody buffer (0.1 M PBS, pH 7.4, containing 0.1% Triton X-100) and incubated overnight at 4°C with mouse monoclonal anti-p53 (DO-1) and rat monoclonal anti-Hsp90α antibody at 50:1 dilution. In addition, all appropriate negative controls without the primary antibodies were run in parallel. Unbound antibodies were removed by centrifuging nuclear preparations at 800 *g *for 3 min at room temperature (22–24°C) followed by three subsequent 5 min washes with shaking (300 r.p.m.) in antibody incubating buffer at room temperature. Next, the bound primary antibody was detected by using species-specific secondary antibody conjugated with different fluorescent dyes at 100:1 dilution in antibody incubation buffer at room temperature for 1 hour. The mouse IgG secondary antibody conjugated with Oregon green was used to detect mouse monoclonal antibody against p53 (DO-1) and the rat IgG secondary antibody conjugated with Texas red was used to detect rat monoclonal antibody against Hsp90α (Molecular Probes, Invitrogen Detection Technologies). The unbound secondary antibody was removed by using three washes as described earlier for primary antibody. After three washes, nuclear fractions were diluted in antibody incubating buffer without Triton X-100 and spread onto a SuperFrost-plus slides. The air-dried slides were then coverslipped with medium containing the nuclear stain DAPI, as before. The immunofluorescence for DAPI, Texas Red and Oregon green was detected with an Axioplan 2 epifluorescent microscope (Zeiss, Thornwood, NY) equipped with appropriate filters. Individual nuclei were visualized at × 20 magnification.

## Results and discussion

### Inhibition of transcriptional reactivation function of p53 with PFTα

**Figure 2:**
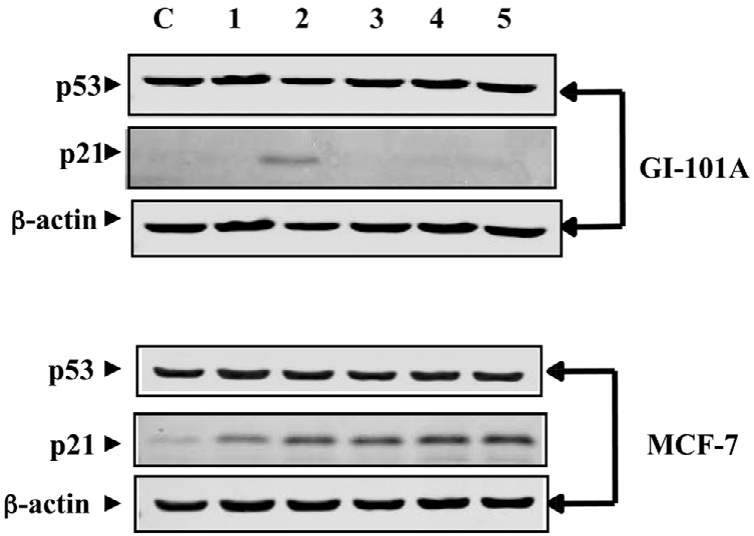
Inhibition of PRIMA-1 mediated transcriptional reactivation function of p53 with pifithrin-α (PFTα). MCF-7 (p53^+/+^) and GI-101A (mut p53) cells were treated with 100 μM PRIMA-1 for 2, 4 and 8 hours (lanes 1, 2 and 3, respectively). Cells were treated with 20 μM PFTα for 6 hours (lane 4) or with 20 μM PFTα for 2 hours followed by PRIMA-1 for 4 hours (lane 5). 20 μg of protein samples of cell lysates were separated by SDS-PAGE (4 to 20% polyacrylamide) and subjected to Western blot analysis with p53 and p21 primary antibodies. The reactive bands were revealed and detected with the Odyssey™ Infrared Imaging System. β-Actin was used as a loading control for protein samples.

We previously reported that PRIMA-1 restored the transcriptional activity of p53 target genes, such as p21^*Waf1*/*cip1*^, in breast cancer cells [18]. The IC_50 _values for PRIMA-1 in MDA-231, GI-101A and MCF-7 cells were determined as 141, 51 and 122 μM, respectively. We selected p21^*Waf1*/*cip1 *^as a marker for measuring the extent of the transcriptional reactivation of p53 by PRIMA-1 at both mRNA and protein levels, because the promoter of the *p21* gene exhibits high occupancy to wild-type p53 protein on its *p53 *binding sites, *in vivo*; it is therefore considered a benchmark for p53-dependent genes [22]. However, p21^*Waf1*/*cip1 *^can also be transcriptionally regulated by p53-independent mechanisms [23,24]. To determine whether the expression of p21^*Waf1*/*cip1 *^is dependent on the restored transcriptional function of p53, cells were treated with PRIMA-1 in the presence and in the absence of PFTα. PFTα is a small molecule that was isolated for its ability to block p53-dependent transcriptional activation [25]. As shown in Fig. 2, treatment of GI-101A cells (mut p53) with 100 μM PRIMA-1 induced the expression of p21^*Waf1*/*cip1 *^at 4 hours. However, treatment of these cells with PRIMA-1 in the presence of 20 μM PFTα resulted in an inhibition of p21^*Waf1*/*cip1 *^expression. No change in the level of p53 protein was observed under these conditions. In contrast, no change in the expression of p21^*Waf1*/*cip1 *^protein was observed when MCF-7 cells (wild-type p53) were treated with PRIMA-1 in the presence of PFTα, confirming the specificity of action of PFTα as an inhibitor of p53-dependent transcriptional function. The lack of inhibition of p21 expression in MCF-7 cells after treatment with PFTα suggests that there is p53-independent expression of p21 in these cells or that MCF-7 cells is not sensitive to the dosage of PFTα used in our studies. Furthermore, the data also show that mutant p53 reactivation by PRIMA-1 results in the transcriptional activation of p53 target genes such as p21^*Waf1*/*cip1*^. However, the exact molecular mechanisms by which this activation occurred are not yet determined. Identification of the molecular targets that are involved in mutation reactivation of p53 by PRIMA-1 is essential for understanding the molecular mechanisms for p53 mutation reactivation and for devising therapeutic strategies aimed at enhancing the use of PRIMA-1 in cancer therapy. It is conceivably possible, for example, that PRIMA-1 affects cellular chaperones resulting in the refolding of mutant p53. In an attempt to identify possible molecular targets involved in mutation reactivation of p53 by PRIMA-1, we used a functional proteomics approach in which cell lysates were co-immunoprecipitated with DO-1 primary antibody directed against p53 after treatment with PRIMA-1 followed by protein partner identification with MALDI-TOF mass spectrometry.

### Identification of Hsp90 as a candidate target for p53 mutation reactivation

**Figure 3:**
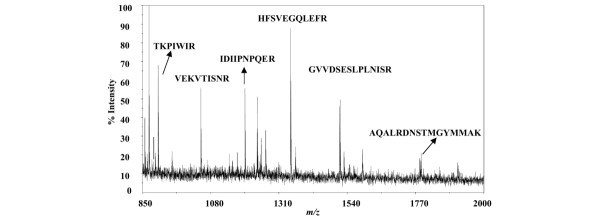
Peptide mass fingerprinting of in-gel tryptic digest of the 90 kDa band generated with a matrix-assisted laser desorption ionization-time-of-flight mass spectrometer. Protein bands indicated by the arrowhead in Fig. 1 were subjected to in-gel digestion and analyzed by mass spectrometry. The tryptic peptides from this band showed the presence of six peptides corresponding to heat shock protein 90 (Hsp90) as one of the proteins that were found in the altered protein-protein interaction pattern of p53 with and without PRIMA-1 treatment.

Figure 1 shows a Coomassie blue-stained gel of proteins co-immunoprecipitated with DO-1 antibody from MDA-MB-231 cells (mut p53) after treatment with 100 μM PRIMA-1 for 4 hours. We chose to resolve proteins by one-dimensional electrophoresis because we were able to observe clearly and reproducibly the separation of protein mixtures, especially that of proteins smaller than 100 kDa. Single bands of polyacrylamide gel slices from SDS-PAGE that are differentially expressed after treatment with PRIMA-1 were excised and subjected to in-gel digestion by trypsin. After digestion, a small portion of the supernatant was removed and analyzed by high-accuracy peptide mass mapping with MALDI. The peptide masses obtained by MALDI analysis were used to search protein databases. We routinely observe peaks that correspond to keratin, presumably resulting from contamination during the handling of gel slices. Such peaks were excluded from our analysis and were occasionally used for internal calibration. Figure 1 focuses on a single band (90 kDa) that is marked by an arrow because it is one of the bands that is distinctly expressed in MDA-MB-231 cells after treatment with PRIMA-1 and co-immunoprecipitated with DO-1 monoclonal antibody. The tryptic digests of the excised bands were subjected to mass fingerprinting with the MALDI-TOF mass spectrum as reported previously [26]. Figure 3 illustrates the MALDI mass spectrum acquired from the peptide mixture resulting from in-gel digestion of the 90 kDa bands shown in Fig. 1. On average, six peptide masses were identified in this spectrum that agreed with the expected peptide masses within a mass tolerance of ± 0.24 Da. The peak *m*/*z *values were used to search the SwissProt Database with the ProFound program. This search resulted in the identification of Hsp90 (Klenow fragment) with a probability score of 1.0 and *Z *score of 1.8, a strong identification of this protein. The sequence coverage of the matched protein candidate (Klenow fragment) was 27%. These results were repeated with multiple co-immunoprecipitation experiments, which all resulted in the identification of Hsp90α as a candidate protein that is differentially expressed after treatment of cells with PRIMA-1.

**Figure 4:**
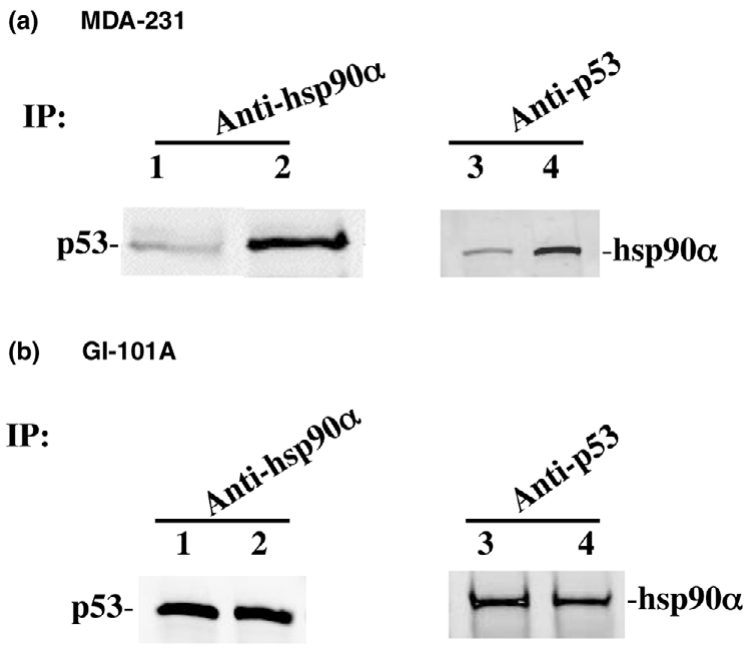
Protein-protein interaction analysis of p53 and the α isoform of heat shock protein 90 (Hsp90α). **(a) **MDA-231 cell lysates from untreated cells (lanes 1 and 3) and cells treated for 4 hours with 100 μM PRIMA-1 (lanes 2 and 4) were immunoprecipitated (IP) with anti-Hsp90α monoclonal antibody and subjected to Western blotting (WB) with anti-p53 (DO-1) monoclonal antibody (lanes 1 and 2) in addition to reciprocal immunoprecipitation with DO-1 and Western blotting with anti-Hsp90α (lanes 3 and 4). **(b) **GI-101A cell lysates from untreated cells (lanes 1 and 3) and cells treated for 4 hours with 100 μM PRIMA-1 (lanes 2 and 4) were immunoprecipitated with anti-Hsp90α monoclonal antibody and subjected to Western blotting with anti-p53 (DO-1) monoclonal antibody (lanes 1 and 2) in addition to reciprocal immunoprecipitation with DO-1 and Western blotting with anti-Hsp90α (lanes 3 and 4).

Because the data collected by peptide mass fingerprinting are sometimes insufficient for the reliable identification of a protein, we further validated and confirmed the identity of Hsp90 protein by immunoblotting analyses. Cells were treated with 100 μM PRIMA-1 for 2, 4 or 8 hours, and protein samples from the whole cell extracts (WCE) and nuclear extracts (NE) were Western blotted with antibodies directed against both the α and β forms of Hsp90 protein. In mammalian cells there are at least two Hsp90 isoforms, Hsp90α and Hsp90β, which are encoded by separate genes. The amino acid sequences of human and yeast Hsp90α are 85% and 90% homologous to Hsp90β, respectively [27]. All known members of the Hsp90 protein family are highly conserved, especially in the amino-terminal and carboxy-terminal regions that contain independent chaperone sites with different substrate specificity [28,29]. To confirm the interaction of p53 with Hsp90α, we performed a co-immunoprecipitation assay with monoclonal Hsp90α antibody on control cells and PRIMA-1-treated cells and subjected the immunoprecipitated proteins to immunoblotting with monoclonal antibody against p53 protein (DO-1). As shown in Fig. 4, both MDA-231 and GI-101A cells exhibited interaction of p53 with Hsp90α protein. The data indicate that p53 interacts with Hsp90α in both breast cancer cells. However, the p53 and Hsp90α protein-protein interaction is different in both cell lines. For example, GI-101A cells show no noticeable changes in protein-protein interaction after treatment with PRIMA-1 (Fig. 4b), whereas more Hsp90α is bound to p53 in MDA-231 cells after treatment with PRIMA-1 (Fig. 4a). This may reflect the phenotype of cells as well as differences in p53 mutations and polymorphism [16].

### Nuclear translocation of Hsp90α after treatment with PRIMA-1

**Figure 5:**
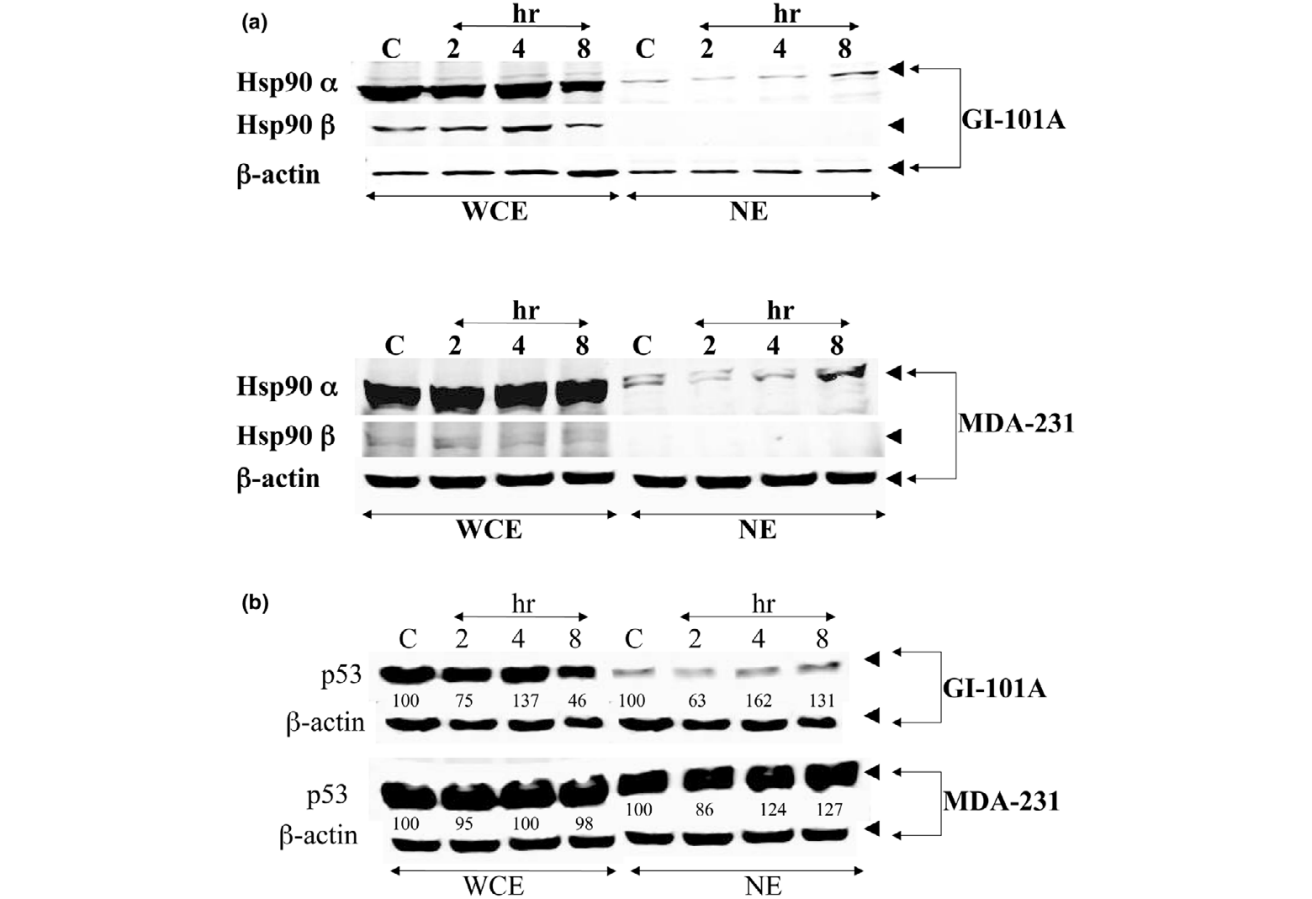
Western blots of heat shock protein 90 (Hsp90) proteins from control and PRIMA-1-treated cells. Cells were treated with 100 μM PRIMA-1 for 2, 4 or 8 hours. 20 μg of protein samples of cell lysates from the whole cell extracts (WCE) and nuclear extracts (NE) of the control (C) and treated samples were separated by SDS-PAGE (4 to 20% polyacrylamide) and Western blotted with antibodies directed against both the α and β isoforms of Hsp90 **(a) **and against p53 **(b)**. β-Actin was used as a loading control. The reactive bands were detected with the Odyssey™ Infrared Imaging System.

The Western blots in Fig. 5a show that both isoforms of Hsp90 are expressed in both cell lines, although in different amounts. The α-isoform is expressed in greater amounts than the β-isoform in samples obtained from both WCE and NE. In addition, Hsp90α was detected in the NE of protein samples obtained from both cell lines. The level of Hsp90α in the NE was increased after treatment of cells with PRIMA-1 for 8 hours. In contrast, the Hsp90β isoform was not detected in the NE of protein samples obtained from either cell line, suggesting that only the α isoform of Hsp90 is translocated to the nucleus after treatment with 100 μM PRIMA-1. The exact mechanism(s) for this differential translocation are not clear yet. However, the intracellular localization of Hsp90 has been investigated under normal and heat-stressed conditions. After heat stress, an increased amount of Hsp90α was detected in the nucleus of cells compared to unstressed cells[30,31]. For nuclear localization to occur, the nuclear localization signal (NLS) is usually required. For Hsp90α, as well as for many other proteins, the nuclear import is recognized by an NLS receptor (a heterodimeric complex) that is composed of importin α and β subunits [32-34]. It has been shown that, under *in vitro *conditions, Hsp90α interacts with importin α [31].

**Figure 6:**
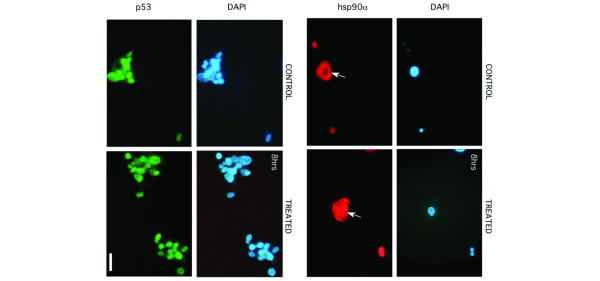
The nuclear localization of the α isoform of heat shock protein 90 (Hsp90α) is enhanced by treatment of cells with PRIMA-1. MDA-231 cells treated with PRIMA-1 were subjected to nuclear isolation. A fraction of the isolated nuclear pellet was fixed in 4% paraformaldehyde, permeabilized in 0.1% Triton X-100 and incubated overnight with anti-p53 and anti-Hsp90α monoclonal antibodies. Nuclear fractions were then immunostained with a secondary antibody conjugated with Oregon green to detect p53 (green) and a secondary antibody conjugated with Texas red to detect Hsp90α (red) before detection by fluorescence microscopy. Nuclei were stained with 4',6-diamidino-2-phenylindole (blue). Normal mouse immunoglobulin G was used as a negative control (data not shown). Arrows mark nuclear staining of Hsp90α. Scale bar, 5 μm.

It is also possible that the nuclear transport of Hsp90α after treatment with PRIMA-1 constitutes part of a selective delivery of restored conformation of p53 to the nucleus. The nuclear transport of the wild-type p53 is also known to be dependent on NLS systems [34]. We therefore investigated whether the observed nuclear accumulation of Hsp90α after treatment with PRIMA-1 was correlated with any changes in p53 protein expression and/or localization. Figure 5b shows the nuclear accumulation of p53 after treatment of cells with 100 μM PRIMA-1 for 2, 4 or 8 hours. The ratio of the integrated absorbance of p53 bands to that of the actin band was used as an index of protein expression in both WCE and NE. It is clearly shown in Fig. 5b that the levels of p53 protein in the NE are much higher after the treatment of both cell lines with PRIMA-1, especially at 8 hours, indicating that the observed increase of Hsp90α protein levels in the NE (Fig. 5a) is correlated with that of p53 protein nuclear accumulation. We observed no change in the level of p53 protein expression or localization when MCF-7 cells were treated with PRIMA-1 (data not shown). These data therefore suggest that the Hsp90 protein is a binding partner to p53 after its conformational restoration by PRIMA-1 and that the α-isoform is involved in the nuclear accumulation of the active form of p53 protein. The enhanced nuclear translocation of Hsp90α was also investigated with immunocytochemistry. Figure 6 shows that Hsp90α is selectively localized in the nucleus of MDA-231 breast cancer cells treated with PRIMA-1 for 8 hours.

Many studies showed that the Hsp90 protein has a major role in the stability of p53 protein and in its translocation to the nucleus. King and colleagues [35] reported that the wild-type p53 protein forms a complex with Hsp90 in the presence of Hop and that Hsp90 may assist p53 import into the nucleus. However, the type of Hsp90 isoform that is actually involved in the nuclear translocation of p53 was not mentioned in that study. Chen and Wang [36] recently reported that the stabilization of p53 conformation after heat shock is associated with its binding to Hsp90 protein and that the phosphorylation of p53 is dependent on the Ataxia-telangiectasia-mutated protein kinase (ATM)-mediated activation of human checkpoint 2 (ChK2) kinase. Although these studies investigated the role of Hsp90 in regulating p53 stability under stressful conditions (heat shock), the present studies confirmed the association between Hsp90 and p53 under non-stressful conditions, because there was no increase in Hsp90 expression in WCE in PRIMA-1-treated cells (Fig. 5b), although a small decrease in WCE of PRIMA-1-treated samples at 8 hours was concomitant with an increase in nuclear translocation of Hsp90α in both cell lines.

Recently, Walerych and colleagues [37] reported that Hsp90α interacts with wild-type p53 protein and that this interaction facilitates the binding of Hsp90α to the p21 promoter in ATP-dependent manner. Murphy and colleagues [38] showed that PFTα inhibits the p53 signaling after interaction with Hsp90α. Thus, both reports [37,38] suggest that the interaction of p53 and Hsp90α enhances the transcriptional transactivation of genes containing p53-binding sites in their promoter region. Our findings can be interpreted as the restoration of the p53-Hsp90α interaction by PRIMA-1, enhancing the nuclear translocation of p53-Hsp90α and reactivating the transcriptional activity of p53.

### Sensitization of breast cancer cells to DNA targeted agents with PRIMA-1

**Figure 7:**
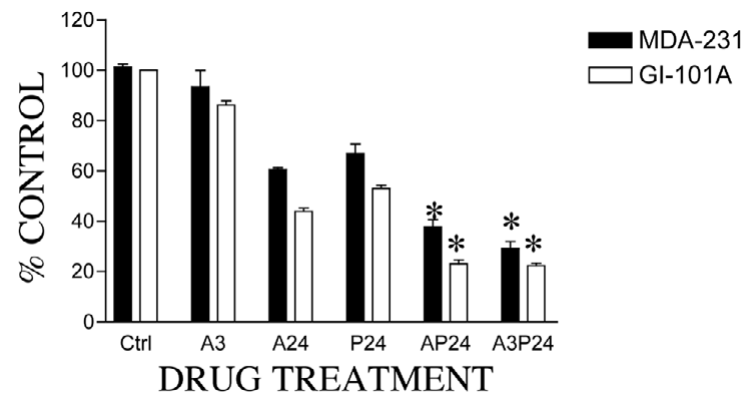
Combination and sequential exposure of cells to PRIMA-1 and adriamycin. Exponentially growing MDA-MB-231 or GI-101A cells were seeded in 10% serum-supplemented RPMI-1640 medium at 10^4 ^cells per well. After 24 hours, cells were treated with 100 μM PRIMA-1 for 24 hours (P24), 0.2 μM adriamycin for 3 hours (A3) or 24 hours (A24), and a combination of PRIMA-1 plus adriamycin for 24 hours (AP24) or adriamycin for 3 hours followed by PRIMA-1 for 24 hours (A3P24). After drug treatment, the cells were reincubated in drug-free medium for a further 3 days and cell survival was determined with the crystal violet assay. Results are means ± SD for quadruplicate determinations (SD<10%) representative of two to four independent experiments; *P*<0.01.

We next examined whether reactivation of the p53 mutation by PRIMA-1 would enhance the cytotoxicity of DNA-damaging agents such as adriamycin *in vitro*. Because many studies have shown that p53 mutations are associated with a decreased sensitivity of tumor cells to many chemotherapeutic agents [3-6,39], it seems reasonable that PRIMA-1, which restores p53 transcriptional activity and enhances its nuclear translocation, would increase the sensitivity of these cells to DNA-targeted agents. Figure 7 shows that simultaneous treatment of human breast cancer MDA-231 and GI-101A cells *in vitro *with 100 μM PRIMA-1 and 0.2 μM adriamycin for 24 hours, or sequential treatment with adriamycin for 3 hours followed by PRIMA-1 for 24 hours, significantly enhances the sensitivity of tumor cells to the drug combination compared with the use of each drug alone. On close examination of the percentage of cell survival after drug treatment of MDA-231 cells, it is clearly shown that adriamycin treatment for 3 hours (A3) had 95% cell survival, compared with only 30% in the presence of PRIMA-1 (A3P24). Treatment of these cells for 24 hours with PRIMA-1 alone produced 75% cell survival. These data suggest that PRIMA-1 enhances adriamycin efficacy and that the drug combination, in this case, is synergistic or supra-additive. Preliminary evidence from our laboratory indicates that the drug combination is also synergistic on a panel of breast cancer cell lines (Tao Wang and S.S.D., unpublished work).

## Conclusion

This study illustrates the use of a functional proteomics approach to identify target molecules that are associated with the reactivation of a p53 mutation by PRIMA-1. Our approach has identified Hsp90α as a partner protein that is associated, in part, with the restoration of p53 transcriptional transactivation function by PRIMA-1. We also showed that the α isoform of Hsp90 protein is associated with the nuclear translocation of p53 protein after treatment of breast tumor cells with PRIMA-1. Our results that PFTα inhibits the induction of p21 expression after treatment with PRIMA-1, and the recent work of Murphy and colleagues [38] indicating that PFTα inhibits the signaling of p53-mediated gene transcription, strongly suggest that PRIMA-1 facilitates the interaction of Hsp90α and the restoration of mutant p53 to wild type followed by the translocation of both proteins into the nucleus. This may result in the transactivation of genes containing p53-binding sites as reported by Walerych and colleagues [37].

## Abbreviations

DAPI = 4',6-diamidino-2-phenylindole; Hsp90α = heat shock protein 90, α isoform; Hsp90β = heat shock protein 90, β isoform; MALDI-TOF = matrix-assisted laser desorption ionization-time-of-flight; NE = nuclear extracts; NLS = nuclear localization signal; PBS = phosphate-buffered saline; PFTα = pifithrin-α; WCE = whole cell extracts.

## Competing interests

The author(s) declare that they have no competing interests.

## Authors' contributions

AR performed cell culture studies, immunoblotting and immunoprecipitation, subcellular fractionation and immunocytochemistry, and participated in drafting the manuscript. MSC participated in immunoblotting studies. XT performed out in-gel enzymatic digestion of separated bands, and mass spectrometry. JEB participated in the protein identification and sequencing. YP participated in the design of the study and drafting of the manuscript. SSD conceived the study, participated in the design of its study and coordination and drafted the manuscript. All authors read and approved the final manuscript.
